# Investigation of Tool Degradation during Friction Stir Welding of Hybrid Aluminum–Steel Sheets in a Combined Butt and Overlap Joint

**DOI:** 10.3390/ma17040874

**Published:** 2024-02-14

**Authors:** Robin Göbel, Stefanie Schwertel, Stefan Weihe, Martin Werz

**Affiliations:** Materials Testing Institute (MPA), University of Stuttgart, Pfaffenwaldring 32, 70569 Stuttgart, Germanymartin.werz@mpa.uni-stuttgart.de (M.W.)

**Keywords:** friction stir welding, tool wear, tool degradation, H13 steel tool, EN AW-6016, DX54D, hybrid tailor welded blanks, dissimilar welding, stripe light projection

## Abstract

Friction stir welding, as a solid-state welding technique, is especially suitable for effectively joining high-strength aluminum alloys, as well as for multi-material welds. This research investigates the friction stir welding of thin aluminum and steel sheets, an essential process in the production of hybrid tailor-welded blanks employed in deep drawing applications. Despite its proven advantages, the welding process exhibits variable outcomes concerning formability and joint strength when utilizing an H13 welding tool. To better understand these inconsistencies, multiple welds were performed in this study, joining 1 mm thick steel to 2 mm thick aluminum sheets, with a cumulative length of 7.65 m. The accumulation of material on the welding tool was documented through 3D scanning and weighing. The integrity of the resulting weld seam was analyzed through metallographic sections and X-ray imaging. It was found that the adhering material built up continuously around the tool pin over several welds totaling between 1.5 m and 2.5 m before ultimately detaching. This accretion of material notably affected the welding process, resulting in increased intermixing of steel particles within the aluminum matrix. This research provides detailed insights into the dynamics of friction stir welding in multi-material welds, particularly in the context of tool material interaction and its impact on weld quality.

## 1. Introduction

The combination of different sheet metals, such as aluminum and steel, is of great interest for lightweight car body design. One possibility for combining the advantages of both metals in a specific and economical way involves so-called tailor welded blanks. Deep-drawing blanks, tailored to the different load zones, are welded together from different alloys and sheet thicknesses prior to forming. As a solid-phase joining process, friction stir welding (FSW) is particularly suitable for welding these dissimilar materials.

In friction stir welding, the workpieces are joined by a rotating tool which is forced through the joint gap. The workpiece material is not liquefied in the process as in classic fusion welding, but is only brought to a plastic state by the friction between the tool and workpiece and the extreme deformation during stirring. As a result, no solidification processes take place, and the additional heavy plastic deformation allows fine grained, high-strength microstructures to be formed in the joint. In addition, the process is suitable for joining dissimilar materials with different melting temperatures, such as aluminum and steel. In order to join aluminum and steel as flat sheets of different thicknesses in such a way that the formability allows for a subsequent deep drawing process, classic overlap and butt joints are often not sufficiently suitable. Overlap welds show limited tensile and fatigue strength due to the bending moment resulting from the overlap and the crack-like incisions, as shown by Kleih [1]. Conventional butt welds, on the other hand, are especially suitable for joining sheets of the same thickness, and they limit the joining interface to the cross section of the thinner sheet.

**Figure 1:**
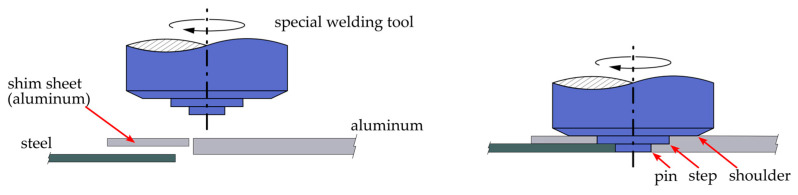
Combined butt and overlap joint between a 2 mm aluminum sheet and a 1 mm steel sheet.

A special joining configuration, developed at the Material Testing Institute in Stuttgart (MPA) [2], allows a sheet made of a high-strength aluminum alloy to be joined to a thinner steel sheet with an increased joining interface. In this novel friction stir welding configuration, the joint consists of a combination of a butt joint on the face and an overlap joint on the top surface of the thinner sheet. The tool used for this kind of joint has a stepped pin (see Figure 1). The peripheral surface of the pin and the face of the step are in contact with steel and aluminum, while the peripheral surface of the step and the tool shoulder are in contact with aluminum only. The step in the aluminum sheet can be made either by milling it down by the thickness of the steel sheet, in this case 1 mm, or alternatively by using a shim sheet of aluminum which compensates for the difference in thickness of the two sheets. In the welding process, the tool mainly stirs the aluminum, which is significantly plastically deformed. The steel is only activated by the relative rotation of the tool. The lateral penetration depth of the tool into the steel, which is set in a range within 0.2–0.6 mm, is referred to as offset.

It is crucial to avoid excessive offset, as this can significantly reduce the strength of the weld joint [3]. Kimapong notes that deeper penetration into the steel leads to increased formation of brittle intermetallic compounds due to greater heat generation [4]. This observation is affirmed by Werz, who documents in his dissertation that brittle fractures occur along intermetallic compounds at offsets exceeding 0.7 mm [5]. Furthermore, Watanabe and Chen have observed an increase in steel fragments scattering into the aluminum matrix at larger offsets, also resulting in a decrease in tensile strength [6,7]. These findings underscore the importance of precise control over the offset to ensure the integrity and strength of weld joints.

**Figure 2:**
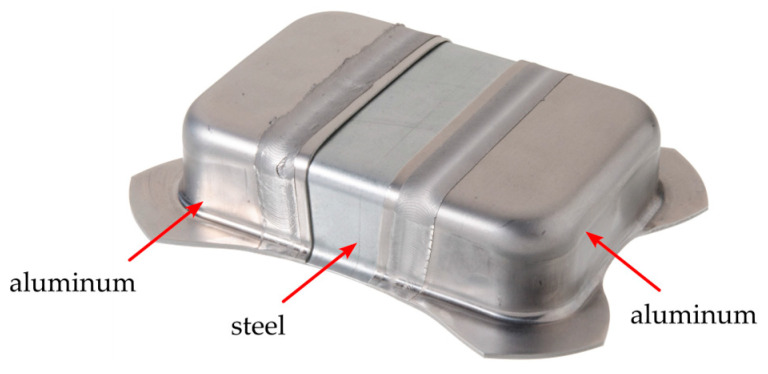
Deep-drawn friction stir-welded aluminum–steel hybrid.

In previous studies, it has been shown that friction stir welding with this special joining configuration, the combined butt and overlap joint, can produce high-quality joints between aluminum and steel sheets. Werz [5] reached strengths of up to 90% of the aluminum base material with this joint during tensile testing transverse to the welding direction. Through a special heat treatment subsequent to the welding process, the tensile strength could even be increased above the strength of the aluminum. Additionally, Nakajima tests confirmed that the joint strength could surpass that of the base [3]. The high strength and formability of the welded sheets allow for a subsequent deep-drawing process (see Figure 2). This makes them especially suitable for the production of hybrid aluminum–steel tailor-welded blanks, which offer a promising approach for the lightweight design of car bodies.

Despite its proven capabilities, laboratory tests indicate that the process, in its current stage of development, exhibits relative instability. Not all welds produce a satisfactory outcome in terms of formability and joint strength. Significant differences in quality have been observed in several welds with identical welding parameters, the same tool, and on the same machine. These are sometimes expressed in the form of visually clearly discernible defects. Individual, visually flawless weld seams burst during forming or revealed significant variation in the maximum elongation during tensile tests transverse to the welding direction.

At the same time, the friction stir welding tool, made from H13 tool steel, exhibited signs of degradation in the form of heavy buildup of workpiece material. Within the first few meters of welding, in particular, the area between the pin and the step became clogged with material.

The formation of material buildup on the tool is therefore analyzed and quantified in this study. Furthermore, the relationship between rapid tool degradation, changing tool geometry, and quality differences is investigated.

The wear of tools in friction stir welding has been investigated in a variety of publications. Even some quantitative studies on the wear of H13 tools exist, such as Hasieber [8,9] on aluminum and Sahlot [10,11] on copper alloys, that observe abrasive wear phenomena. Extensive abrasive wear also occurs to a greater extent when welding metal–matrix composites, which contain particularly hard particles [12]. For this reason, alternative, harder tool materials are being investigated in this area in particular [13]. A tool that is harder in relation to the matrix material is found to reduce the wear rate [14]. However, the excessive buildup of material on the friction stir welding tool in the form occurring in this case has not yet been described.

## 2. Materials and Methods

**Figure 3:**
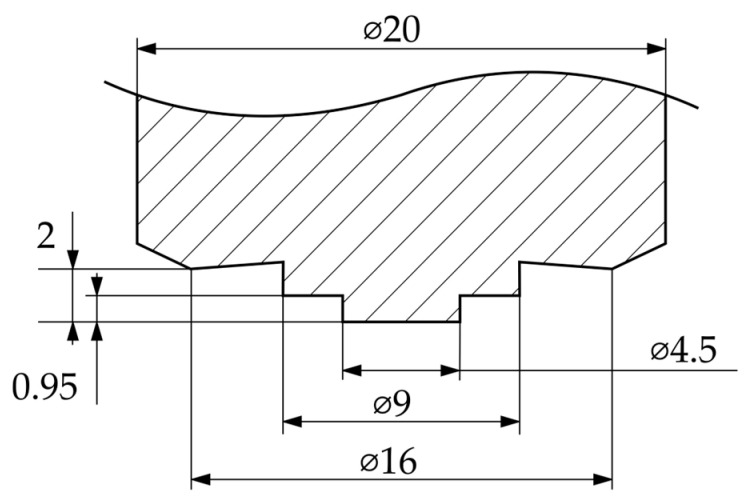
Tool geometry used for friction stir welding of 2 mm aluminum sheets to 1 mm steel sheets.

In this study, sheets of EN AW-6016 aluminum with a thickness of 2 mm in T4 state and 1 mm thick sheets of DX54D steel were chosen as the workpieces. Another 1 mm thick shim sheet of EN AW-6016 was used to equalize the thickness between the two sheets. The friction stir welding tool was made of hot-working steel H13, through-hardened after turning, and tempered to 52 + 2 HRC (see Figure 3 for the geometric details of the tool and Table 1 for the alloy composition of the materials used).

All welds in this study were performed with an ESAB Legio 3ST friction stir welding machine, which can perform linear welds with a maximum axial force of 25 kN and a feed speed up to 4000 mm/min. Welding can be either force- or position-controlled; in this study, welding was exclusively performed under position control, i.e., with a constant plunge depth of the tool. A total of 17 welds, each with a length of 450 mm, were performed with a tool that was new and unused at the beginning, adding up to a total weld length of 7.65 m. All welds were performed with the welding parameters listed in Table 2.

**Figure 4:**
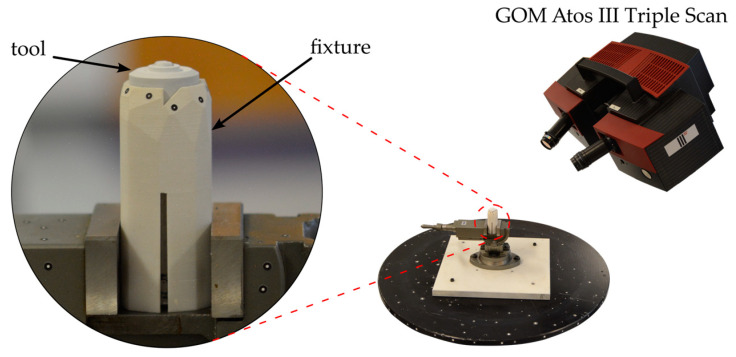
Measurement setup for 3D scanning of the tool with GOM Atos III Triple Scan.

After each weld, the tool was removed from the welding machine, the condition was first documented photographically, and weighing was carried out using a Mettler Toledo AT400 precision scale. After cooling to room temperature, the tool was then 3D-scanned using the GOM Atos III Triple Scan, which covers a measuring point distance of 0.019 mm within the measuring volume of 60 × 45 × 30 mm. To prevent reflections during stripe light projection and thus improve the scan result, the tool was sprayed with a thin layer of titanium oxide powder, which was removed with alcohol prior to the following weld. In order to ensure the angularly accurate allocation of the individual scans, the otherwise rotationally symmetrical tool was marked on the shaft before the start of the study and precisely aligned for the 3D scan in a special fixture (see Figure 4).

The surface models generated from 3D scans could be directly compared with the scan of the unused tool using the ATOS Professional Software (GOM Software 2019). This comparison resulted in differential surface models that graphically illustrated adhesions or abrasions on the used tool.

## 3. Results and Discussion

**Figure 5:**
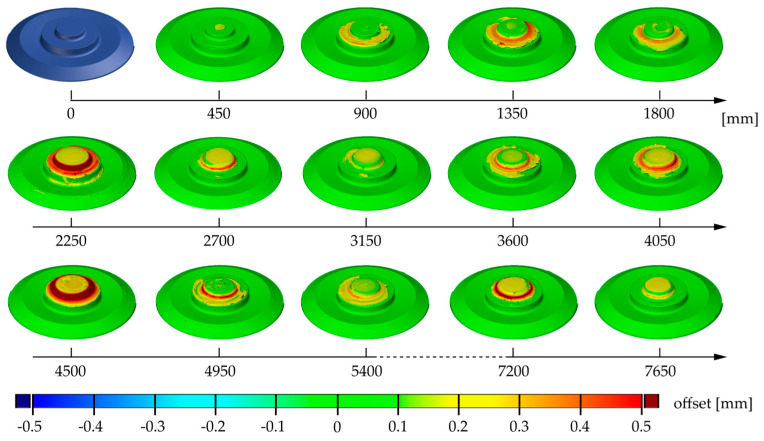
Three-dimensional scanning images of unused and used FSW tools, colored according to the surface comparison to the unused tool.

In Figure 5, the 3D scans of the unused and used tools at different stages are shown. The coloration of the tool from 450 mm to 7650 mm illustrates the surface comparison of the 3D scans taken at that point compared to the scanned geometry at 0 mm. The comparison images are primarily useful for localizing and semi-quantitatively assessing the signs of degradation on the tool.

After 900 mm of welding, material accumulated significantly around the pin. The thickness of the buildup material in this area then continued to increase until it dropped again to a very low level at 2700 mm. Up until 4500 mm, however, the deposit built up continuously to another maximum. This cycle was repeated one more time within the measurement series. Minor material adhesions were also increasingly visible on the pin’s end face.

However, no changes to the tool geometry could be seen on the tool shoulder, which was only in contact with the aluminum during the process. Furthermore, it was noticeable that the surface models did not show abrasive wear at any point.

**Figure 6:**
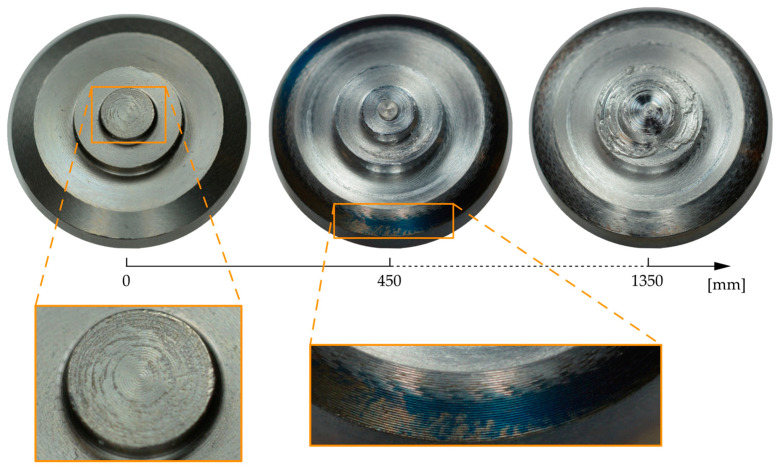
Macroscopic images of the tool throughout the degradation study.

Figure 6 shows macroscopic images of the shoulder and pin of the tool throughout the degradation study. On the surface of the unused tool (0 mm), marks from the turning process can be seen in the form of slight spiral patterns in the shoulder area, as well as microscopic pits on the end face of the pin.

After the first weld of 450 mm, an aluminum-colored coating was visible on all surfaces that were in contact with the workpiece during the process. This coating was initially so thin that it was not visible in the surface comparison shown above. In some places on the outer bevel of the shoulder, an aluminum coating after had not developed 450 mm, so the base material of the tool was still visible. The base material H13 showed a dark blue coloration in these areas, which, according to the annealing colors for steel [18], indicates that temperatures around 300 °C prevailed here during the welding process. After 1350 mm of welding, the material accumulations already shown in the 3D scans were clearly visible in the area between the pin and the step. The material buildup was so significant that the originally sharp pin contour was no longer clearly identifiable.

**Figure 7:**
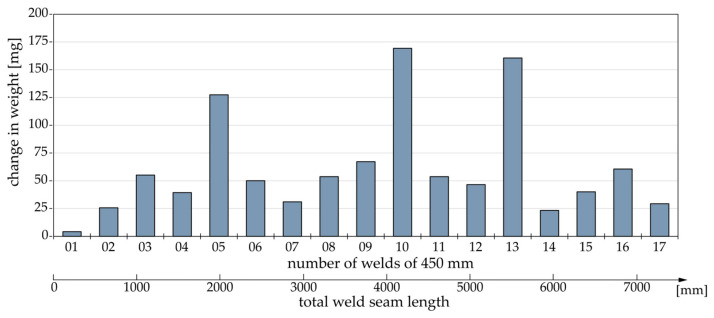
Respective mass difference of the tool compared to the unused tool.

Plotted over the number of the welds performed, Figure 7 shows the respective mass difference of the tool compared to the unused tool. Given that the 3D scans suggested minimal, if any, material removal relative to the buildup, the mass differential can be considered equivalent to the mass of the adhered material. The cyclic adhesion of material to the tool observed in the 3D scans and macroscopic images can also be observed quantitatively in the adhesion mass. At welded lengths of 2250 mm, 4500 mm, and 5850 mm, the adhesions exhibited local maxima, reaching 209%, 277%, and 263% of the average adhesion mass throughout the study.

**Figure 8:**
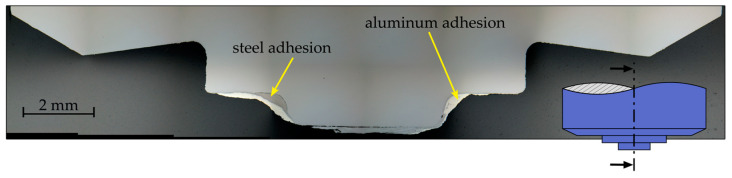
Cross section of another tool after 5465 mm welded length of EN AW-6016 and DX54D, showing aluminum and steel buildup in the step and pin area.

The cross section of another tool of the same geometry and material as that used in the degradation study (Figure 8) after 5465 mm of welded length of EN AW-6016 and DX54D, also showed severe material buildup in the step and pin areas. This cross section allows conclusions to be drawn about the composition of the adhesions. In the area to the left of the pin at the transition between the pin and the shoulder, as well as on the end face of the pin, the buildup of steel could be seen. Aluminum deposits could be seen to the right of the pin and on top of the steel deposit to the left of the pin. The adhering of workpiece material was limited to the designated areas, and the tool shoulder area remained free of heavy material buildup.

**Figure 9:**
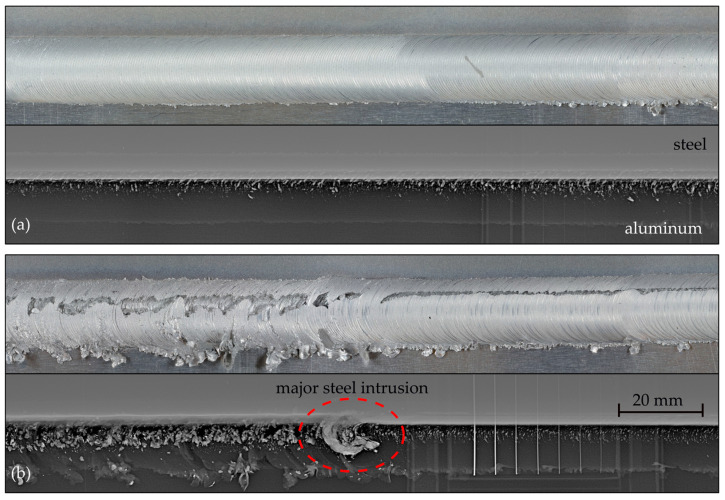
Photographs and X-ray images of (**a**) weld No. 3 at 1160 mm and (**b**) weld No. 6 at 2570 mm of welded length.

Figure 9 shows photographs as well as X-ray images of welds No. 3 (a) and No. 6 (b) at 1160 mm and 2570 mm of welded length, respectively. Despite the same welding parameters, differences in seam quality are clearly visually noticeable. While weld No. 3 produced an optically clean seam, the seam in weld No. 6 had surface defects along its full length and an extensive, frayed flash. Over the remaining 15 welds, the weld quality also proved to be visually very inconsistent, with frequent surface defects.

The X-ray images also showed clear differences between the two seams in the form of differing steel inclusions, which appeared as light gray fringe on the aluminum–steel interface. In weld no. 6, there was also one single major steel intrusion. This intrusion cannot be seen visually, although the pattern of the surface defects changed at that point from coarse and irregular to more even.

**Figure 10:**
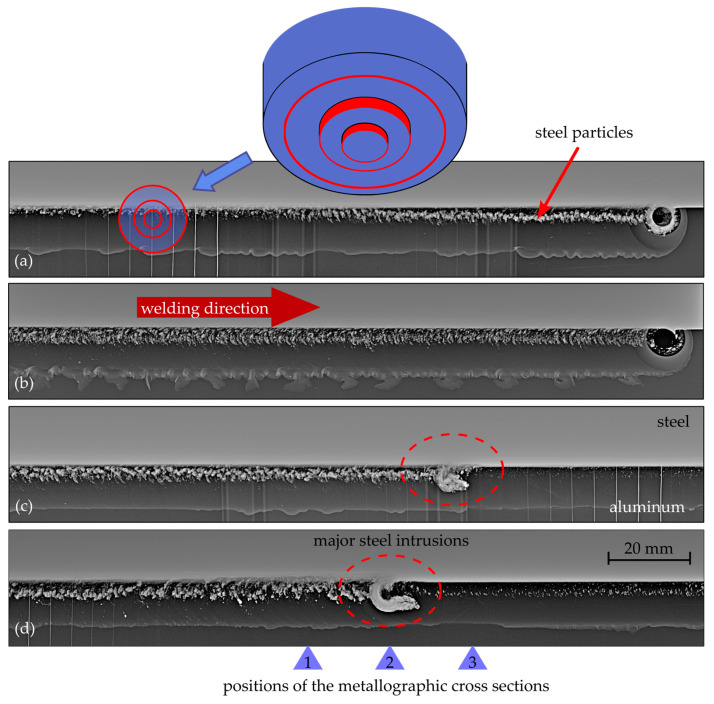
X-ray images of friction stir welds made with identical tools and welding parameters. Images (**a**,**b**) show varying degrees of steel particles protruded into the aluminum. Images (**c**,**d**) highlight different steel intrusions, with (**d**) displaying a prominent hook-shaped feature. Red rings indicate tool dimensions.

Further detailed examinations of the welds using X-ray images are shown in Figure 10. Radiation intensity and exposure time were selected so that the aluminum part of the seam would be clearly visible, while the steel was barely penetrated. The red rings represent the relevant tool diameters. The inner ring is the pin rubbing against the steel with its peripheral surface. And the outer rings represent the tool’s step and shoulder. The welding direction was from left to right. The steel particles stirred into the aluminum are clearly visible as fringes protruding into the aluminum. This fringe of steel particles clearly differed in the various welds, and also within individual weld seams. In some cases, there was little agitation, and in others, like in (a), the steel particles appeared more as a line seemingly separated from the steel sheet. Another notable detail in the radiography is the hook-shaped steel intrusion in the lower image. Further back in the weld seam, i.e., to the left of the intrusion, a comparatively high level of intermixing of steel particles into the aluminum is visible. On the right, in the subsequent part of the seam, the aluminum contains noticeably fewer and finer particles.

**Figure 11:**
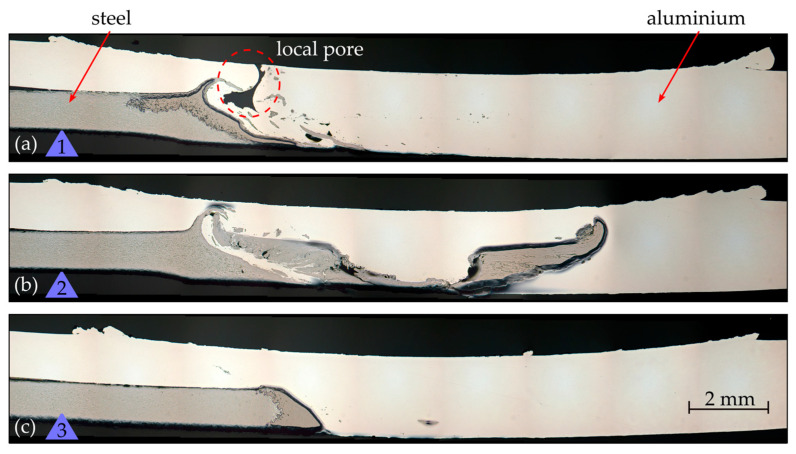
Cross sections of the aluminum steel weld (see location in Figure 10d) at (**a**) 20 mm before the steel intrusion, (**b**) exactly the location of the steel intrusion, and (**c**) 20 mm behind it.

The cross sections in Figure 11 show exactly the location of the conspicuous major steel intrusion in Figure 10d, as well as locations 20 mm before and 20 mm behind it. The part on the left side that appears darker is the steel. The right part, which also overlays the steel on the left, is the aluminum. The flashes on the left and right of the aluminum indicate where the tool was engaged. In cross section 1 (a), before the steel intrusion, it can be seen that the edge of the steel was significantly flattened in the process and that most of the steel particles stirred into the aluminum were located near the seam root. There were only few smaller particles in the upper area of the stirring zone. In addition, a pore is visible, which was not visible in the visual inspection nor in the radiography. Therefore, it can be assumed that this is not a wormhole, but a local, single pore in the microsection plane. At the location of the steel intrusion (cross section 2 (b)), there was a massive accumulation of steel at mid-height in the weld. It is noticeable that the steel part roughly corresponded to a negative impression of the tool. In the follow-up of the steel intrusion (cross section 3 (s)), only a single steel particle stirred into the aluminum remained visible. Otherwise, there was a sharp division between aluminum and steel.

The investigations show that, even within less than 1 m of the weld seam, severe deviations in the geometry of the tool occurred due to adhering workpiece material. This led to significant optical differences in weld quality, as the appearance of the weld seam fluctuated between a clean look and one showing severe surface defects. Moreover, these variations contributed to considerable inconsistency in the offset at which the tool laterally penetrated the steel, which depends on the extent of material buildup. The effect of this occurrence could be observed when examining the weld via radiography and cross sections in the form of different types of steel inclusions. The gradually increasing buildup on the tool led to deeper penetration into the steel, thereby stirring more steel particles into the aluminum. In the radiograph, it can also be seen how, after around 2.5 m, the accumulated workpiece material detached from the tool and remained in the weld seam. However, only the steel portion of the adhered material was visual. Subsequently, adhesion built up again over another 2 m of welding.

As highlighted in the introduction, the accurate control of the offset is crucial in dissimilar FSW of aluminum and steel to maintain the integrity and strength of the joints. The scattering of larger steel pieces into the aluminum matrix and the formation of brittle intermetallic compounds, which occur due to increased heat generation at larger offsets, significantly compromise mechanical strength [4,6,7].

Consistent with these observations, a study by Kaushik, which involved welding with different tool rotation-to-feed rate ratios at a consistent offset of 0.5 mm, revealed similar effects. An increase in heat input led to a greater intermixing of steel particles, contributing to a reduction in the mechanical strength of the welds [19].

Furthermore, accurate modeling of the complex contact conditions in friction stir welding simulations, through well-calibrated contact models between the workpiece material and the tool, is crucial for precisely calculating thermomechanical responses. Various approaches, including Smoothed Particle Hydrodynamics (SPH), Computational Fluid Dynamics (CFD), and Coupled Eulerian–Lagrangian (CEL) methods, as exemplified by Shishova and Wang [20,21], utilize the diverse capabilities of these modeling techniques to accurately depict frictional and heat transfer-related contact behavior. Analytical models have also been developed, providing comprehensive analyses of the generated heat and mechanical responses [21]. A commonality among these approaches is the assumption of constant tool geometry conditions, which, as our findings illustrate, does not align with reality due to cyclic material buildup on the tool. Varying geometrical contact conditions, as observed in this study, should have a significant impact on simulation results.

The phenomenon of material buildup on the tool bears similarities to the buildup of cutting material observed in machining processes, known as buildup edge formation. This behavior involves a cyclic pattern consisting of formation, growth, and detachment, and occurs within specific ranges of (low) cutting speeds [22,23]. Factors such as temperature and roughness of the tool, as well as the general inclination for adhesion between the tool and workpiece, significantly influence the buildup of material on the tool [23]. In the case of welding EN AW-6016 aluminum and DX54D steel with an H13 tool, it would be relevant to investigate the tribological system between these three materials to understand the critical value at which the friction coefficient results in material adhesion. Possible preventative measures used to prevent buildup edge formation include deviation from critical parameter sets (feed rate/rotational speed), coatings to prevent adhesion, texturing of the tool surface, and the use of coolants, although the latter may not be practical in friction stir welding. Varying the speed and feed rate is also not readily feasible in friction stir welding, as these parameters have a great influence on the quality of the weld, such as the formation of intermetallic compounds at the aluminum–steel interface as well as microstructure formation in the stir zone.

It should be considered that the tool was completely cooled down at the beginning of each weld due to the intermediate time-consuming measurement. Furthermore, it can be assumed that the repeated reinitiation of the welding process, i.e., every 450 mm, had an influence on the degradation phenomena on the tool, an issue with this type of wear investigation which is also mentioned by Sahlot [10]. The results of this investigation and, in particular, the frequency at which the buildup on the tool develops and detaches, therefore cannot be transferred to a continuous weld without further ado.

Additionally, the identification of cyclic behavior from 3D imaging and buildup mass measurements presumes that the frequency of these occurrences is not less than the 450 mm sampling interval, a presumption that is supported by radiographic imaging.

## 4. Conclusions

This study explores tool degradation during friction stir welding of aluminum and steel sheets in a combined butt and overlap joint, and its effect on the resulting weld seam is investigated.

The main findings are as follows:

1. When welding EN AW-6016 aluminum and DX54D steel in combined butt and overlap joint configuration using a tool made of H13, tool degradation occurs in the form of material buildup on the portion that is in direct contact with the steel during the process.
2. The material adhering to the tool accumulates over several welds, reaching a maximum after a total welded length of 1.5 to 2.5 m before detaching. This cycle of buildup and detachment recurs throughout the welding process.
3. The effect of the enlarged tool pin caused by material adhesion is evident in the welding process and, consequently, in the manufactured weld, as confirmed by radiographic imaging and cross-sectional analysis of the weld seam. Increased adhesion to the tool causes a higher degree of steel particle intermixing within the aluminum matrix.
4. Different processing regimes, in terms of largely varying offset conditions due to the material adhesion, occur even when the same tool, machine, materials, and parameters are used.
5. The robustness of the process is not yet sufficient for an industrial-scale production, indicating a need for further optimization.

The investigations presented here reveal the phenomenology of the effect. The mechanisms that generate or stabilize the different regimes, and how or why the adhesions are detached above a certain size, are not yet fully understood. Furthermore, the influence of the varying process regimes on the mechanical properties is also not yet known. Considering these findings, it is recommended that future research focus on these aspects. Such research should include different tool materials and different workpiece materials, alongside a systematic mechanical analysis of the welds in their different states.

Initial welding experiments utilizing ceramic and coated tools suggest that tool degradation due to excessive material buildup can be mitigated, potentially leading to a more stable and consistent welding process.

## Figures and Tables

**Table 1:** Alloy composition of EN AW-6016 T4, DX54D, and the tool material H13 according to DIN EN 573-3, DIN EN 10346, and DIN EN ISO 4957 [15,16,17].

EN AW-6016 (AlSi1.2Mg0.4)	Si	Fe	Cu	Mn	Mg	Cr	Zn	Ti
	[wt.%]	[wt.%]	[wt.%]	[wt.%]	[wt.%]	[wt.%]	[wt.%]	[wt.%]
Chemical Composition [15]	1.0–1.5	0.50	0.20	0.20	0.25–0.6	0.10	0.20	0.15
DX54D	C	Si	Mn	P	S	Ti		
	[wt.%]	[wt.%]	[wt.%]	[wt.%]	[wt.%]	[wt.%]		
Chemical Composition [16]	≤0.12	≤0.50	≤0.60	≤0.10	≤0.045	≤0.30		
H13 (X40CrMoV5-1)	C	Si	Mn	Cr	Mo	V	W	
	[wt.%]	[wt.%]	[wt.%]	[wt.%]	[wt.%]	[wt.%]	[wt.%]	
Chemical Composition [17]	0.35–0.42	0.80–1.20	0.25–0.50	4.8–5.5	1.20–1.50	0.85–1.15	-	

**Table 2:** Welding parameters used for all welds throughout this study.

	Rotational Speed (1/min)	Feed Speed (mm/min)	Tool Offset (mm)	Heel Plunge Depth (mm)	Tool Tilt Angle (°)
Process Parameters	1200	700	0.2	−0.18	2

## Data Availability

Data are contained within the article.
